# Supplementary material for: The Construction and Exploration of a Comprehensive MicroRNA Centered Regulatory Network in Foxtail Millet (Setaria italica L.)
Source: Front Plant Sci. 2022 May 6;13:848474. doi: 10.3389/fpls.2022.848474 (PMC9121102; doi:10.3389/fpls.2022.848474)
Supplement: Supplementary file 1 [file Data_Sheet_1.zip › Sit-miR319_Seita.9G064600.1_1005_TPlot.pdf]

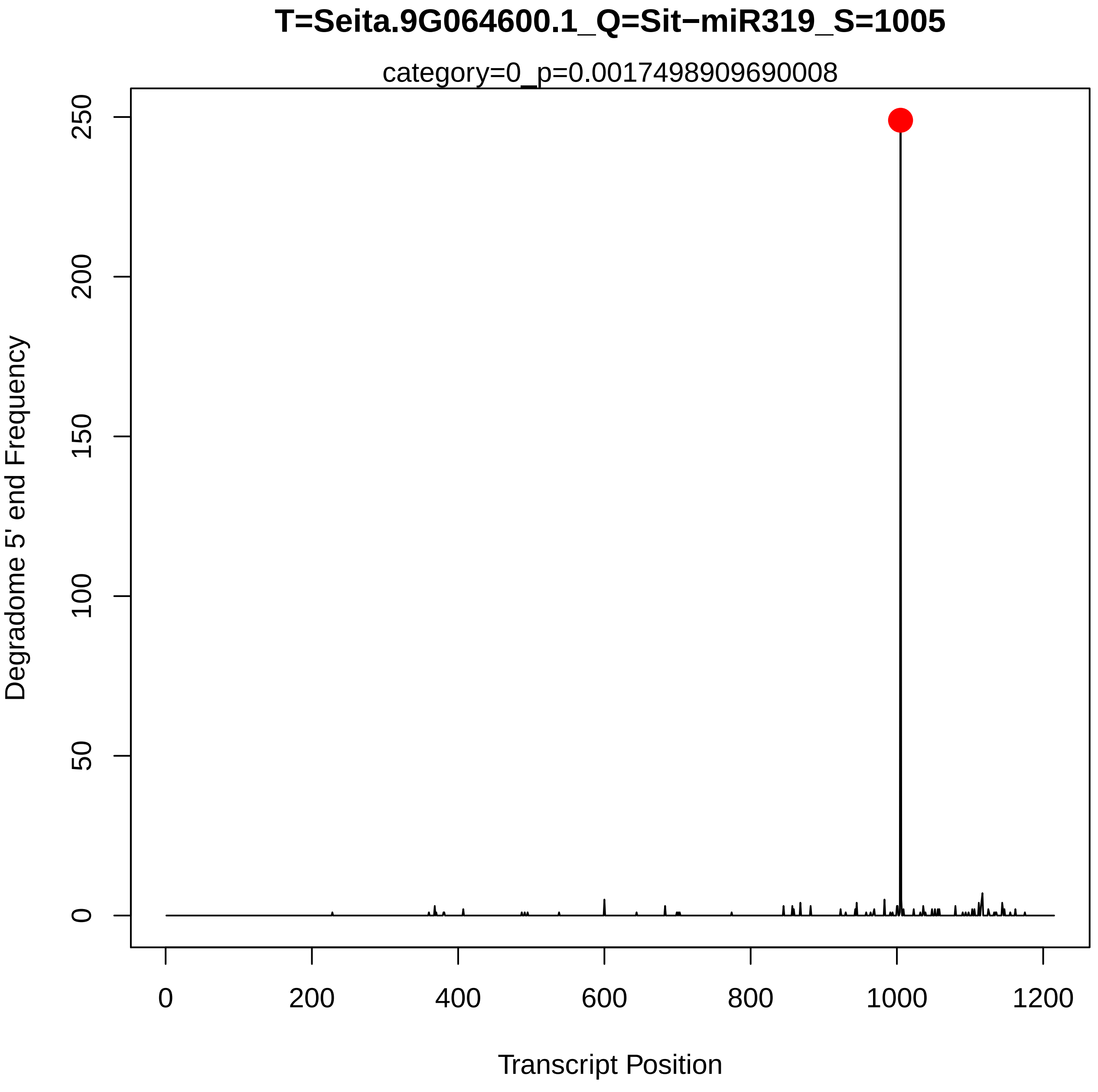
T=Seita.9G064600.1_Q=Sit−miR319_S=1005
category=0_p=0.0017498909690008
Degradome 5' end Frequency
0
50
100
150
200
250
0
200
400
600
800
1000
1200
Transcript Position
